# JBMRPlus: Special Issue on Rare Bone Diseases 2019

**DOI:** 10.1002/jbm4.10218

**Published:** 2019-08-09

**Authors:** Eileen M. Shore, Maurizio Pacifici

**Affiliations:** ^1^ Departments of Orthopaedic Surgery and Genetics Perelman School of Medicine at the University of Pennsylvania Philadelphia PA; ^2^ Translational Research Program in Pediatric Orthopaedics, Division of Orthopaedic Surgery The Children's Hospital of Philadelphia Philadelphia PA

## Introduction

Rare genetic diseases are rare because the genes mutated in, and responsible for, these disorders are poorly tolerated developmentally and physiologically. Selection against these changes thus makes these mutations rare in the population, but also identifies the corresponding genes as critical to cell function and organism well being and growth. This special issue of *JBMR Plus* presents examples of our growing knowledge and new perspectives of musculoskeletal biology and of cell and tissue processes impacting skeletal health that are being gained through the study of rare diseases.

Several rare bone diseases are discussed in this issue, each caused by genetic mutations that impact bone and cartilage in multiple ways, including altering signals that direct the induction of endochondral ossification (FOP), increased proliferation of immature osteoblasts (melorheostosis), fibrous dysplasia of bone (MAS), bone fragility and impaired mineralization (OI, HPP, FGF23‐mediated hypophosphatemia), and tooth and enamel development (in a mouse model of decreased FGF signaling).

Increased understanding of the underlying causes of these conditions is leading to advances in treatments. Additionally, several of the articles emphasize the importance of multidisciplinary care in treating these conditions. Since the effects of gene mutations that cause rare conditions are often not limited to a single clinical consequence, more attention is being given to gaining a more complete understanding of both the full pathological effects of the disease and system‐wide effects beyond the primary clinical concern. This approach is not only providing new directions to improve overall health and well‐being of those affected, but also revealing previously unrecognized direct and indirect consequences of the underlying mutations.

The case report by Corsi et al. describes an example of the rarest of the rare – a case of neonatal lethal multi‐organ McCune‐Albright Syndrome (MAS) caused by somatic gain‐of‐function mutation of the *GNAS* gene. The clinical consequences of somatic mutations are highly dependent on when during development the mutation occurs and where the mutation is distributed among various tissues. In the presented case, clinical effects are closely correlated with the prevalence of the mutation in relevant tissues. A perhaps unanticipated but illustrative finding was skeletal bone effects, despite the absence of the frequently MAS‐associated fibrous dysplasia of bone and a low frequency of mutated cells in bone. The authors highlight and discuss how indirect effects of the mutation on bone nonetheless can result in critical impact and clinical consequences.

Osteogenesis imperfecta (OI) is a condition of defective bone formation, most often caused by mutations in the COL1A1 or COL1A2 genes which code for collagen type I alpha proteins. However, mutations in at least 18 other genes have been discovered to cause forms of OI, and have provided new insight into the mechanisms of collagen function, structure, and processing in bone. The review by Tauer et al. focuses on the recent clinical and translational opportunities for OI that are being developed following this improved understanding of the underlying mechanisms of OI pathogenesis.

Following the determination that activating *ACVR1* mutation is the cause of fibrodysplasia ossificans progressiva (FOP), a rare genetic disorder of heterotopic ossification (HO), significant advances are being made in understanding the mechanisms leading to disease pathology along with advances toward treatments. However, an area that has received relatively little attention is the pain associated with episodes of disease activity (flare‐ups) and the impact of flare‐up dependent and independent pain on emotional health and pain perception. These relationships and their impact on overall quality of life for people with FOP were investigated by Peng et al., exploring an issue that has relevance to many other rare and common diseases.

As for the study on FOP, gaining a more complete understanding of the impact of a disease was undertaken by Jha et al. through a natural history clinical study of melorheostosis, a skeletal dysostosis of cortical bone overgrowth. A systemic analysis of this condition, beyond its most prominent clinical effects, has identified frequent sensory deficits, focal nerve entrapment, and pain, and is providing opportunities to improve overall health, well‐being, and clinical management of patients with this condition.

Deficient alkaline phosphatase activity in hypophosphatasia (HPP) causes impaired skeletal mineralization that results in fractures and impaired fracture healing. Treatment of HPP with asofotase alfa (AA) improves skeletal health and growth. However the bone marrow edema (BME) that has been reported in juvenile forms of HPP has no established therapy. Schmidt et al. report the effects of the bone anabolic drug teriparatide on BME on two patients with HPP.

Fibroblast growth factor (FGF) signaling is an important cell pathway that mediates a range of cell and tissue functions and involves a diverse family of signaling proteins and their receptors. Studies of rare hypophosphatemias and associated skeletal defects led to the identification of FGF23, which is produced primarily in bone, as a key regulator of phosphate homeostasis. An excess of FGF23 activity is now recognized to be responsible for a number of conditions. Imel et al. review FGF23‐assciated disorders and hypophosphatemia, and discuss the biomedical and clinical studies that have led to development of the FGF23 antibody burosumab for the treatment of X‐linked hypophosphatemia and the potential for this drug to treat other conditions characterized by increased FGF23 activity.

FGF signaling and FGF23‐mediated hypophosphatemias are frequently associated with dental complications, but it remains unclear how the FGF signaling pathway regulates tooth development and impacts tooth morphology and enamel quality. Marangoni et al. have developed an informative transgenic mouse model in which the FGF pathway inhibitor Sprouty4 is overexpressed to investigate the impact of FGF signaling on odontogenesis.

Patient support and advocacy organizations are critically important partners for research and clinical advances for rare disorders. Information is included for the Rare Bone Disease Alliance (RBDA) and its member organizations which have been instrumental in providing education about and advocacy for rare bone conditions.

In sum, this special issue of *JBMR Plus* highlights the significant progress being made in the field of rare musculoskeletal diseases and underlines the importance of these efforts to clarify the pathogenesis of these diseases in greater and greater detail and in so doing, identify ever more effective and safe treatments and patient care modalities.

RARE BONE DISEASE ALLIANCE

The Rare Bone Disease Alliance is a program of the Osteogenesis Imperfecta Foundation, www.oif.org

The Alliance Director is Charlene Waldman, waldmancharlene234@gmail.com

The Rare Bone Disease Alliance (rbdalliance.org), originally created in 2006 as a patient advocacy network, is a coalition focused on educating medical professionals, expanding research and assisting patients and families affected by rare bone diseases.

The Alliance encourages professional, medical and scientific societies to expand their educational programs on rare bone disease and organizes its own meetings and workshops. In September 2018 the Alliance organized the conference Mechanistic and Therapeutic Advances in Rare Skeletal Diseases. Meeting summary and abstracts in the journal JBMRPlus at https://onlinelibrary.wiley.com/doi/10.1002/jbm4. 10136.

Alliance participants include rare bone disease physicians and scientific thought leaders, the Rare Bone Disease patient (RBDPN) organizations and pharmaceutical companies working in the rare bone field.

RBDPN Organizations: Fibrous Dysplasia Foundation, International Fibrodysplasia Ossificans Association (IFOPA), Lymphangiomatosis & Gorham's Disease Alliance, Lymphatic Malformation Institute, The MHE Research Foundation, Osteogenesis Imperfecta Foundation. The Osteopetrosis Society, Soft Bones: The U.S. Hypophosphatasia Foundation, XLH Network.

